# Further learning of clinical characteristics and imaging manifestations of nonketotic hyperglycemic hemichorea

**DOI:** 10.1111/1753-0407.13543

**Published:** 2024-04-07

**Authors:** Xiaoyu Wang, Yuting Zhang, Fan Yang, Suqing Bao, Lijun Duan, Xia Jiang

**Affiliations:** ^1^ Department of Endocrinology and Metabolism Tianjin First Central Hospital Tianjin China; ^2^ Department of Neurology Tianjin First Central Hospital Tianjin China; ^3^ Department of Neurosurgery Tianjin First Central Hospital Tianjin China

**Keywords:** hemichorea, nonketotic hyperglycemia, type 2 diabetes mellitus

## Abstract

**Objective:**

To summarize the clinical characteristics and imaging manifestations of patients with nonketotic hyperglycemic hemichorea (NH‐HC) and to explore the possible pathogenesis, diagnosis. and treatment of the disease in order to improve the understanding of this disease and avoid misdiagnosis.

**Methods:**

Retrospective analysis was performed on the case data of five patients with NH‐HC admitted to our hospital in recent years. The patients were treated in the department of endocrinology, department of neurology, and department of neurosurgery in our hospital, respectively. Meanwhile, relevant literatures were consulted for further learning.

**Results:**

NH‐HC is usually presented as a triad of nonketotic hyperglycemia, lateral chorea, and typical imaging manifestations of head magnetic resonance imaging or computed tomography, but the clinical manifestations are not the same, and imaging features may also be different, presenting a diversified trend in clinical practice. All five patients were given glucose‐lowering drugs and improved with or without combination of drugs to control symptoms of chorea.

**Conclusion:**

NH‐HC is a rare complication of diabetes, characterized by hyperglycemia and hemichorea. How to identify the extreme situation and make fast judgment is a top priority. Timely and correct control of blood glucose is the key to the treatment, and when necessary, application of dopamine receptor antagonists in patients with combination therapy can accelerate improvement of the clinical symptoms. The prognosis of NH‐HC is good, the clinician should strengthen comprehensive understanding of this disease to avoid missed diagnosis or misdiagnosis and enable patients to get more timely and effective treatment.

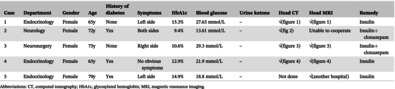

## Graphical abstract

Highlights

- Nonketotic hyperglycemic hemichoea (NH‐HC) is a rare complication of diabetes. Because it may involve the departments of endocrinology, neurology, and neurosurgery, it is often missed diagnosed or misdiagnosed.
- NH‐HC has a range of relevant clinical and imaging features, but the clinical heterogeneity is large, and not all patients have typical findings.
- We analyzed the clinical manifestations and imaging features of the disease through five related cases to provide some ideas for the future clinical diagnosis and treatment of NH‐HC. How to quickly and accurately identify this disease in clinical work and treat it as soon as possible is the top priority.

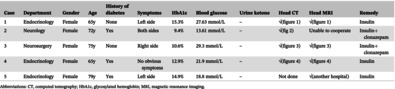

## 1 INTRODUCTION

Clinically, patients with chorea are more commonly seen in the department of neurology or neurosurgery. Chorea can be caused by cerebrovascular disease, Huntington's disease, genetic disease, poisoning, and other reasons involving the basal ganglia region. Most patients with hyperglycemia with or without ketosis are referred to the department of endocrinology. But there is a rare chorea caused by hyperglycemia, namely nonketotic hyperglycemic chorea. Due to different symptoms and appearances, patients may be treated in neurology, neurosurgery, endocrinology, or even the emergency department or intensive care unit. As an interdisciplinary disease, clinicians should pay attention to it so as to avoid missed diagnosis or misdiagnosis. Chorea or athetosis caused by nonketotic hyperglycemia has been reported abroad,^1^ and domestic reports have gradually increased in recent years.^2^, ^3^ This paper summarizes the characteristics, diagnosis, and treatment of patients with nonketotic hyperglycemic chorea in recent years admitted to our hospital and reviews the literature.

## 2 CASE SUMMARY

A retrospective analysis was performed on five patients with nonketotic hyperglycemic chorea recently diagnosed and treated in our hospital, who were respectively hospitalized in endocrinology department (admitted in the year of 2018, 2020, 2020, respectively), neurology department (admitted in the year of 2019) and neurosurgery department (admitted in the year of 2018).

### 2.1 Case 1

**FIGURE 1:**
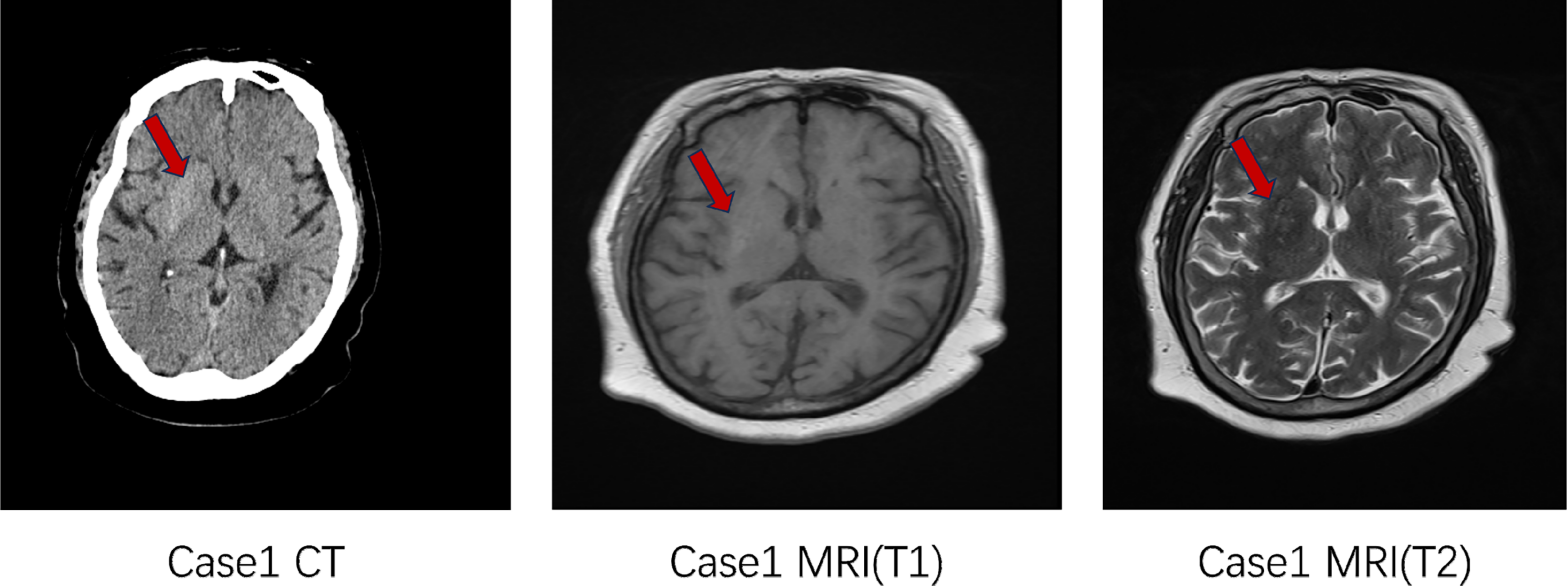
Imaging manifestation of case 1. CT, computed tomography; MRI, magnetic resonance imaging.

Female, 65 years old, denied any history of diabetes. The patient was hospitalized in the endocrinology department with the chief complaint “Left upper limb involuntary movement for 2 days, blood glucose rise for 1 day.” Glycosylated hemoglobin (HbA1c) at admission was 15.3%, with a random blood glucose 27.63 mmol/L and a negative urine ketone body. The main symptoms include involuntary movements around the left side of the mouth, dance‐like movements on the left side (characterized by aimless, irregular, and rapid involuntary movements), involuntary bending, adduction, abduction and torsion of the joints of the upper limb, external display of the lower limb, constant flexion or extension of the toes and ankles, lack of coordination of muscle tension in the left lower limb, slightly reduced muscle tension in the left upper limb, and normal muscle tension in the right limb. Head computed tomography (CT) (Figure 1) suggested patchy high‐density shadows in the right basal ganglia, and head magnetic resonance imaging (MRI) considered long T1 and equal T2 signal shadows in the right basal ganglia.

### 2.2 Case 2

**FIGURE 2:**
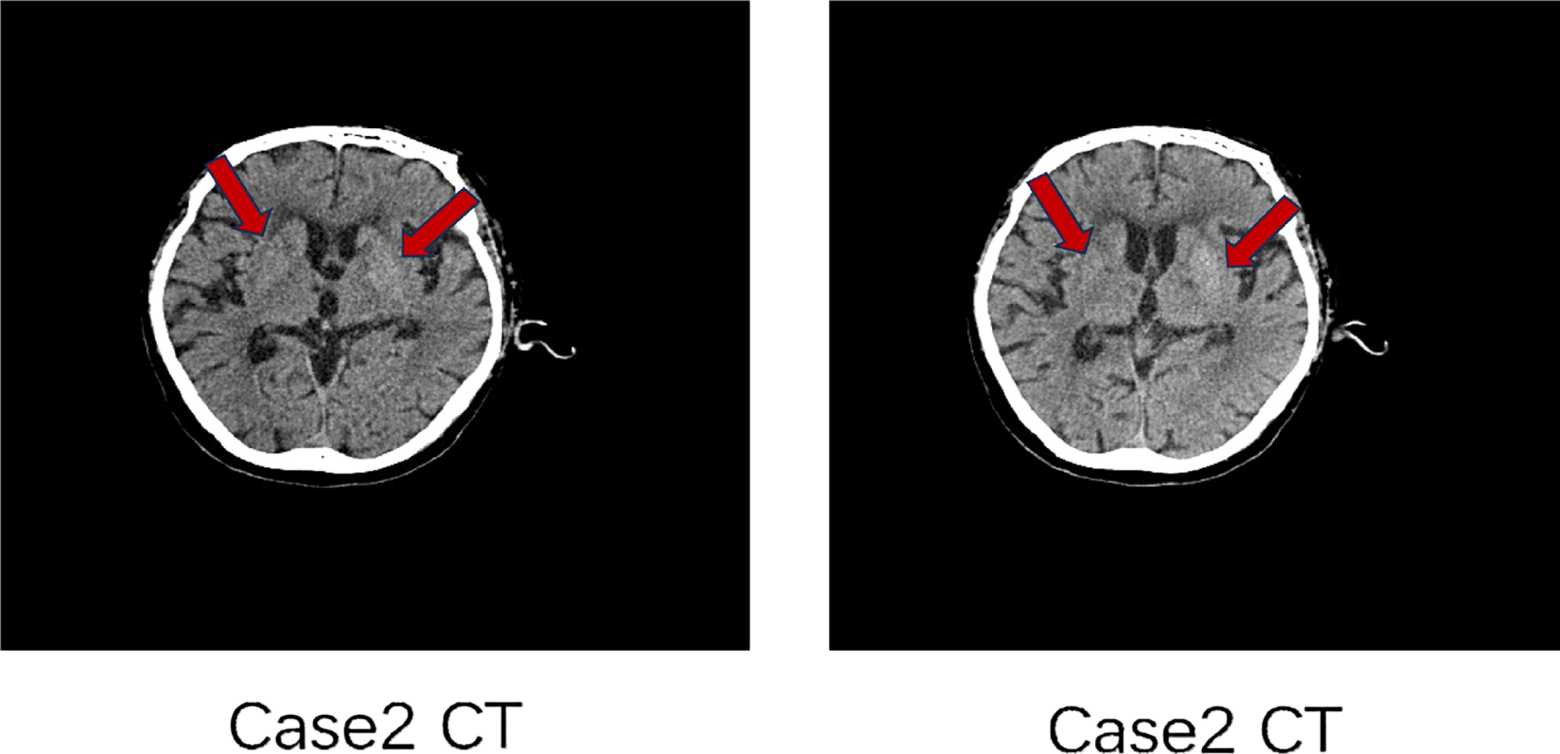
Imaging manifestation of case 2. CT, computed tomography.

Female, 72 years old, with a history of diabetes for more than 20 years (the patient could not describe the exact time). The patient was hospitalized in the neurology department with the chief complaint “Involuntary movements of the head and limbs for 2 weeks.” HbA1c at admission was 9.4%, with a random blood glucose 13.61 mmol/L and a negative urine ketone body. There were no obvious causes of involuntary movements of the face and limbs, manifested as shrug shoulders and waist twisting, kneeling knees and kicking and protruding chest and abdomen, and other involuntary and irregular activities, with large joints as the center; sometimes there were symptoms such as winking, bared teeth, nodding neck, etc., aggravated in activities and mental tension, and disappeared after sleep. In case 2 (Figure 2), due to the uncooperative head MRI examination because of bilateral chorea, only head CT examination was done, with high‐density shadow in the left basal ganglia region and slightly high‐density shadow in the right basal ganglia region considered. It is recommended to improve head MRI examination and consider hyperglycemia‐associated chorea.

### 2.3 Case 3

**FIGURE 3:**
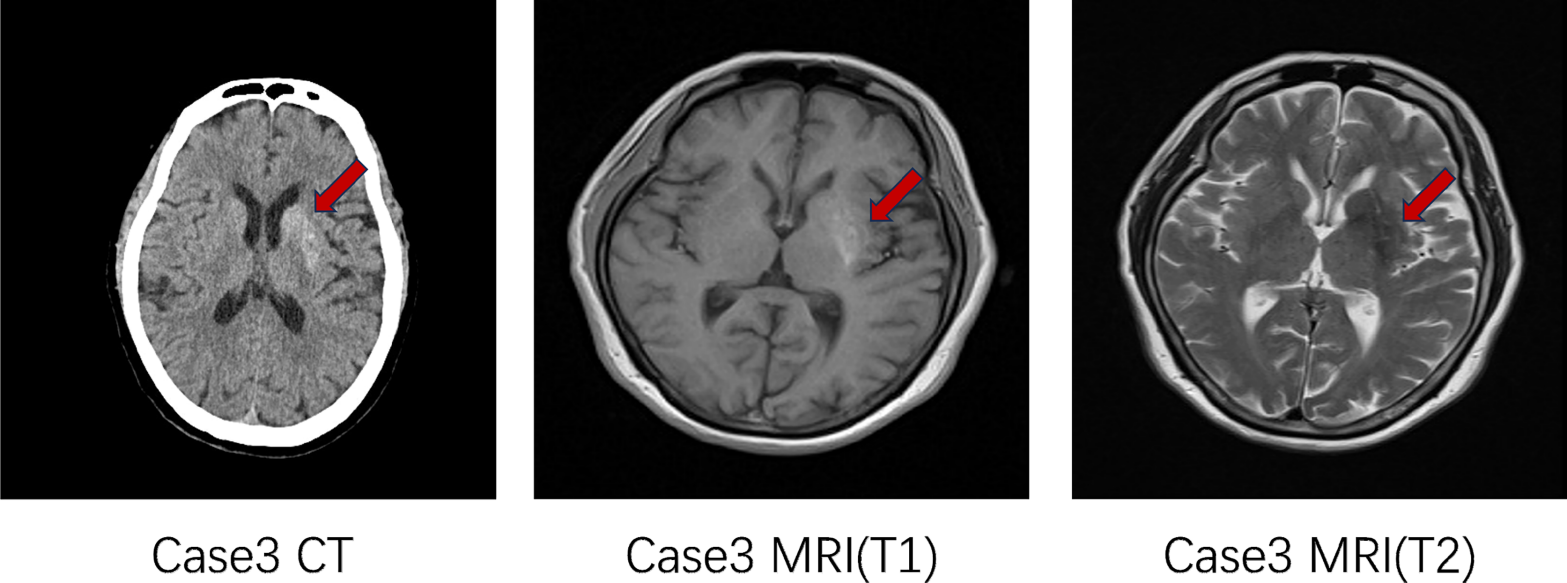
Imaging manifestation of case 3. CT, computed tomography; MRI, magnetic resonance imaging.

Female, 75 years old, denied any history of diabetes. The patient was hospitalized in the neurosurgery department with the chief complaint “Involuntary movements of right limbs for two weeks.” HbA1c at admission was 10.6%, with a random blood glucose 29.3 mmol/L and a negative urine ketone body. There were no obvious causes for involuntary movement of the right limb, accompanied by numbness of the right forearm, nausea, and vomiting. The vomit was stomach contents. The muscle strength of the left limb was grade 5, and the muscle strength of the right limb was grade 5‐, and the muscle tone was normal. Head CT (Figure 3) of case 3 with right hemichorea began to be considered cerebral hemorrhage in the left basal ganglia region, and MRI examination of the posterior head suggested that nonketotic hyperglycemia with hemichorea was considered for diagnosis.

### 2.4 Case 4

**FIGURE 4:**
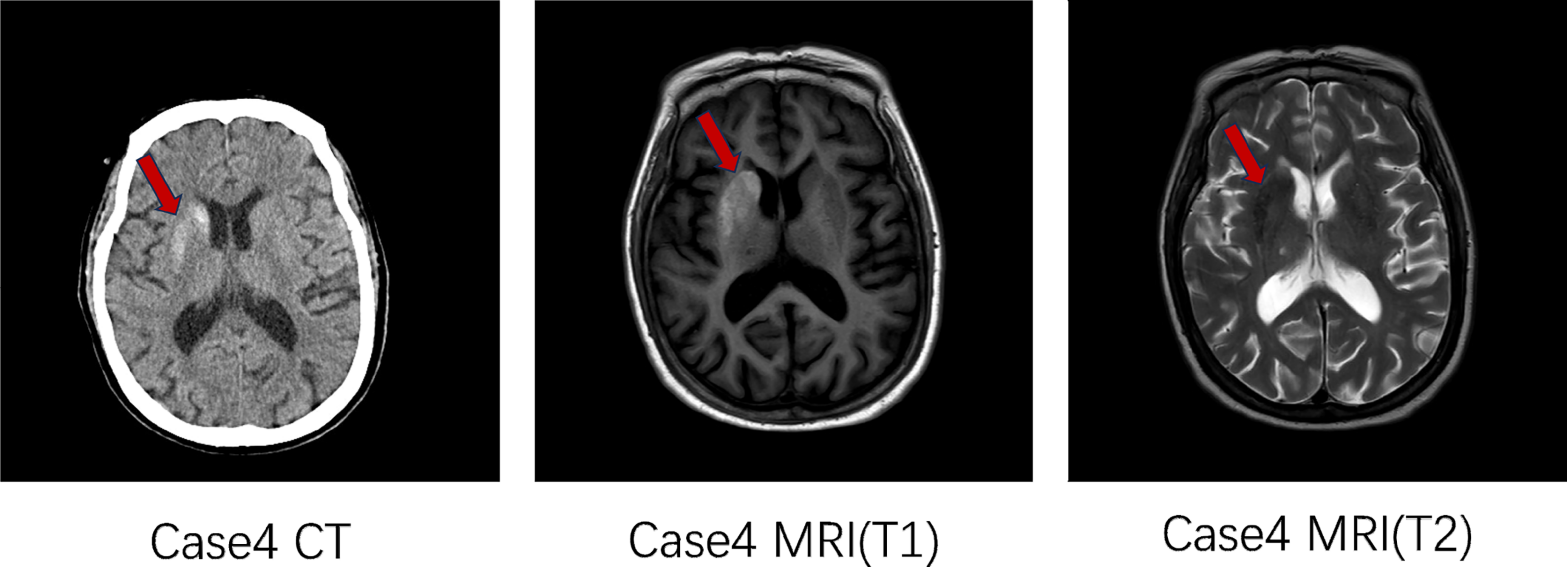
Imaging manifestation of case 4. CT, computed tomography; MRI, magnetic resonance imaging.

Female, 72 years old, with a history of diabetes for 16 years. The patient was hospitalized in the endocrinology department with the chief complaint “Blood glucose rises for 16 years.” HbA1c at admission was 12.9%, with a random blood glucose 21.9 mmol/L and a negative urine ketone body. This patient did not have abnormal limb movement. Head CT (Figure 4) showed abnormal density of basal ganglia region, considered hyperglycemic hemichorea, head MRI presented short T1 signal on the right side of the putamen, and caudate nucleus, also considered hyperglycemic hemichorea, consistent with the CT results.

### 2.5 Case 5

Female, 79 years old, with a history of diabetes for 7 years. The patient was hospitalized in the endocrinology department with the chief complaint “Blood glucose rises for 7 years, left upper limb and left lower limb involuntary movement for 1 week.” HbA1c at admission was 14.9%, with a random blood glucose 18.8 mmol/L and a negative urine ketone body. The patient in case 5 developed an involuntary tic in the left upper limb and left lower limb before admission, no weakening of muscle strength, no nausea or vomiting, no headache or dizziness, accompanied with poor blood glucose control. The head MRI in another hospital (before admission) suggested that hyperglycemic hemichorea (the patient cannot provide CT/MRI images but had the report of them) should be considered, and the disease was also considered after consultation in the neurology department of our hospital.

### 2.6 General information

All five patients were middle‐aged and elderly women, aged 65, 72, 75, 65, and 79 years old respectively, with an average age of 71. Three of them had a history of diabetes for many years, and the other two were newly diagnosed diabetes patients after admission. Diseases that may cause similar clinical symptoms such as basal ganglia cerebrovascular disease, hepatolenticular degeneration, Huntington's disease, carbon monoxide or alcohol poisoning, and echinocytosis were excluded.

Among the five patients (Table 1), except for 1 patient (case 4) with no obvious symptoms(imaging diagnosis), the other four cases were acute onset of involuntary limb movement without obvious cause, including three cases of unilateral limb involuntary movement, two cases of left side, one case of right side, and one case of bilateral limb involuntary movement. Symptoms include involuntary movements around the mouth, aimless, irregular, rapid involuntary movements of the limbs, involuntary alternating flexion and extension, adduction, abduction and torsion of the joints, as well as facial winking, bared teeth, nodding and turning the neck, etc. Each patient is different; symptoms are aggravated during activity and mental tension and disappear after asleep. Patients were conscious, giving fluent answers. The random blood glucose of the five patients at the onset were 27.63 mmol/L, 13.61 mmol/L, 29.3 mmol/L, 21.9 mmol/L, and 18.8 mmol/L, and HbA1c was 15.3%, 9.4%, 10.6%, 12.9%, and 14.9%, respectively. Their urine ketones were negative and only two had occasional weak positive ketone during the course of the disease.

**TABLE 1:** Summaries of five patients.

Case	Department	Gender	Age	History of diabetes	Symptoms	HbA1c	Blood glucose	Urine ketone	Head CT	Head MRI	Remedy
1	Endocrinology	Female	65y	None	Left side	15.3%	27.63 mmol/L	–	√(figure 1)	√(figure 1)	Insulin
2	Neurology	Female	72y	Yes	Both sides	9.4%	13.61 mmol/L	–	√(fig 2)	Unable to cooperate	Insulin+ clonazepam
3	Neurosurgery	Female	75y	None	Right side	10.6%	29.3 mmol/L	–	√(figure 3)	√(figure 3)	Insulin+ clonazepam
4	Endocrinology	Female	65y	Yes	No obvious symptoms	12.9%	21.9 mmol/L	–	√(figure 4)	√(figure 4)	Insulin
5	Endocrinology	Female	79y	Yes	Left side	14.9%	18.8 mmol/L	–	Not done	√(another hospital)	Insulin

Abbreviations: CT, computed tomography; HbA1c, glycosylated hemoglobin; MRI, magnetic resonance imaging.

All five patients were treated with insulin and oral drugs for hyperglycemia, and two were treated with clonazepam (case 2 and case 3). The clinical symptoms of three patients (cases 1, 2, and 5) were significantly improved after treatment, and the drug was gradually reduced without recurrence. The symptoms of one case (case 3) were slightly improved, but the patient and his family strongly requested that he be discharged automatically and go back to the local hospital for further treatment. Unfortunately, we did not follow up successfully because we could not keep in touch with the patient.

## 3 DISCUSSION

The incidence of diabetes is increasing year by year.^4^ As is known to all, the obvious rise of blood glucose may cause complications of multiple organs and various aspects, which are mostly chronic complications of diabetes caused by long‐term poor blood glucose control, whereas diabetic ketosis is a relatively common acute complication of diabetes in clinic. Hemichorea is caused by contralateral extrapyramidal diseases, with a low incidence, accounting for about 2.8% of movement disorders. Hemichorea is most commonly seen in cerebrovascular diseases, or caused by infection, poisoning, metabolic disorders, trauma, etc. However, hemichorea is also relatively rare clinically due to nonketotic hyperglycemia. Nonketotic hyperglycemic chorea is a relatively rare acute complication of diabetes with low clinical incidence, which is not easy to recognize, and some of it occurs in patients with no previous history of diabetes (just like some of our cases), which is very easy to be misdiagnosed. Through the diagnosis and treatment of the five clinical cases in this paper, combined with review of a variety of literature, we will have a systematic further learning about nonketotic hyperglycemic chorea.

Since 1960, hemichorea associated nonketotic hyperglycemia was first reported by Bedwell.^5^ With the increasing number of clinical cases reported, further studies have been conducted. Nonketotic hyperglycemic hemichorea (NH‐HC) is also being recognized. This is a group of clinical syndromes characterized by nonketotic hyperglycemia, hemichorea symptoms, and T1WI hypersignal in MRI of the contralateral basal ganglia of the limb. NH‐HC is more common in elderly diabetic patients with poor blood glucose control, among whom there are also some newly diagnosed diabetic patients who deny the history of diabetes, and most of them are female. According to incomplete statistics, the incidence of this disease is <1/100000. With the increasing incidence of diabetes, hemichorea associated with nonketotic hyperglycemia is also gradually seen in clinical cases. Oh^6^ and partners meta‐analyzed the clinical data of a total of 53 cases reported by various countries. This meta‐analysis collected the data from 53 patients with nonketotic hyperglycemia from different countries showed a male‐to‐female ratio of 1:1.76, mean age >70 years old, and more than 90% are Asian. At the onset of the disease, the average blood glucose level was 26.75 mmol/L, the average HbA1c level was 14.4%, and the plasma osmotic pressure was 305.9 mmol/kg. Among the 53 patients analyzed by them, there were 6 patients with bilateral chorea, 47 patients with unilateral chorea, 14 patients with facial chorea, and 3 patients with unilateral limb chorea. Lee^7^ has also reported that NH‐HC is more common in middle‐aged and elderly women with poor blood glucose control, especially in Asian women. In China, Zhang Benshu, director of Tianjin Medical University General Hospital, first reported four cases^8^ in 2003, including one male and three female, with an average age of over 70 years old, with similar clinical manifestations and imaging features. In our paper, the characteristics of five cases of this disease recently diagnosed and treated in our hospital are consistent with those reported in our country and abroad. Head imaging examination indicated that head CT showed early high density in the head of the caudate nucleus and putamen on the opposite side of the lesion, which could disappear within a short time; MRI showed high signal in T1‐weighted images and slightly low signal in T2‐weighted images, and no significant changes in the signal after several months. Hemichorea refers to the acute onset of the disease, involving unilateral and/or bilateral limb involuntary movements, clinical manifestations of rapid, unbalanced, irregular, aimless dance‐like movements and facial abnormalities such as eyebrow squeezing, eye teasing, pouting, and tongue stretching. Most of the attacks occur when awake, can be aggravated when excited, most can be relieved after rest, some can be temporarily disappeared after asleep. However, hemichorea was mostly reported in the previous literature, but bilateral chorea was also reported recently.^9^ NH‐HC ‐hemiballismus rarely occurs in children. But there had been reported an adolescent boy (14‐year‐old) with recent diagnosis of type 1 diabetes who presented with hemichorea and brain imaging finding. The patient was injected insulin for treatment and also prescribed valproic acid to control his symptoms.^10^ Among the cases diagnosed in our hospital, there was one patient with bilateral involuntary movement, and the other three patients with hemichorea. Two on the left side and one on the right side. there was also one case that had no apparent symptoms but had an apparent imaging manifestation, also diagnosed as NH‐HC, indicating that the clinical manifestations of this disease were inconsistent and presented a diversified trend.

The exact pathophysiology and imaging characteristics of nonketotic hyperglycemia are not yet known, but hyperglycemia, hemichorea, and imaging characteristics (contralateral putamen, lentiform nucleus, and caudate nucleus showed high signal in T1‐weighted images on MRI. In contrast, head CT showed high‐density shadow in the head of the caudate nucleus, putamen, and globus pallidus on the opposite side of the lesion.^11^) and other related characteristics constitute a group of clinical syndromes, With the improvement of the disease, imaging changes are reversible. Previous articles^12^ had reported that hemichorea associated with nonketotic hyperglycemia can be roughly summarized into five types: (a) classic, most of which are of this type; (b) no hyperglycemia, but symptoms and imaging changes; (c) nonketotic hyperglycemia with symptoms but no imaging changes; (d) nonketotic hyperglycemia with imaging changes but no symptoms (just like our case 4); and (e) nonketotic hyperglycemia, bilateral symptoms, and imaging changes (showed as case 2). Currently, it is rare in clinical practice, and in‐depth study of this disease can help us improve the corresponding diagnostic level in clinical work.^13^ Moreover, the clinical characteristics and imaging manifestations of patients with this disease are not absolutely consistent. Some patients have clinical manifestations but not necessarily accompanied by imaging changes, and some patients may have typical imaging changes but no obvious clinical symptoms. One case reported a 75‐year‐old black female patient with a medical history of depression, hypertension, and diabetes. She was presented to the emergency department with a chief complaint of involuntary movements of her right arm and leg for 2 days duration. The CT presenting with NH‐HC demonstrated basal ganglia abnormalities with negative MRI findings.^14^ Therefore, it is more difficult to identify clinically suspected patients with chorea associated with nonketotic hyperglycemia, and it is easier to be misdiagnosed. Moreover, long‐term imaging follow‐up may provide further help for the understanding of this disease. Recent retrospective imaging analysis also suggested that head CT of patients showed increased striatum density on one or both sides, which may involve putamen, lateral part of globus pallidus, or caudate nucleus, and the shadow of decreased or increased striatum density could disappear after reexamination. MRI showed high signal on T1WI and low signal on T2WI of the contralateral lentiform nucleus. MRI review showed no change in signal on T1WI and T2WI of the affected lentiform nucleus.^15^ The caudate nucleus, putamen, and globus pallidus are collectively known as striatum anatomically, which is the main lesion site of chorea. It is the lesion of cholinergic and gamma‐aminobutyric neurons in striatum that leads to the hyperactivity of dopaminergic neurons, thus leading to the occurrence of chorea.^16^ In imaging features, putamen was the first and most commonly involved, and caudate nucleus and globus pallidus were also involved.^17^, ^18^, ^19^, ^20^ A study conducted in 2020 reported that MRI was roughly 16.5% more sensitive in detecting NH‐NC than CT, as MRI readings were consistent with findings of basal ganglia lesions in 95.33% of NH‐HC patients, whereas CT reported a positive finding in 78.86% of these patients.^21^ Of the three regions, the most commonly involved was putamen (78.6%,99/126), followed by caudate nucleus (47.6%, 60/126) and globus pallidus (27.8%, 35/126).^21^ The results were consistent with those of MRI studies in 153 patients showing the frequency of involvement in the order of putamen (94.1%, 144/153), caudate nucleus (64/153), and globus pallidus (43/153). Besides that, a case report also showed that from 18F‐fluorodeoxyglucose positron emission tomography (PET)/CT marked hypometabolism in the basal ganglia contralateral to the side with hemichorea. The metabolic dysfunctions may lead to nonketotic hyperglycemic chorea. The index case demonstrated severe glucose hypometabolism in the striatum and adds to the other reported differential diagnoses for striatal hypometabolism.^22^ Regarding the formation mechanism of imaging findings, studies have shown that there may be the following viewpoints^23^, ^24^, ^25^, ^26^: a local cerebral ischemia or metabolic disorders, (b) glial cell hyperplasia, (c) patchy bleeding caused by metabolic disorders, and (d) reversible calcium salt or some unknown substance deposition, etc. Lai et al^18^ performed a biopsy on a patient with NH‐HC and found that only mild astrocyte hyperplasia and vacuole formation occurred in the lesion site, and there was no deposit of iron or calcium. Shan et al^19^ examined a patient and found mild ischemic changes accompanied by hypertrophic astrocyte hyperplasia. In a recent report, a 55‐year‐old female from Sri Lanka presented with involuntary movements of the left upper limb and lower limb for 2‐week duration. She had a history of diabetes but did not control very well. The scan of the brain showed hyperdensity in the right‐side caudate nucleus, lentiform nucleus, and globus pallidus, suggesting a diagnosis of diabetic striatopathy. This case report was unique as the patient was on anticoagulation, and the hyperdensity in the brain parenchyma in such a patient was easy to be misinterpreted as intracerebral hemorrhage rather than diabetic striatopathy (DS). We need to pay attention in clinical work to avoid misdiagnosis.^27^ Recently, a patient with DS who presented solely with subacute cognitive decline without involuntary movements, and cranial CT showed bilateral high density in the basal ganglia. In contrast, susceptibility‐weighted imagingshowed microhemorrhages in the right caudate nucleus head. After 1 week of treatment, including glycemic control, the patient showed significant improvement in cognitive function, and a repeat cranial CT showed improved hyperdensity in the right basal ganglia region.^28^ In these reports, the symptoms and imaging findings of patients were varied, suggesting clinical heterogeneity. At present, no conclusion has been reached. Previous studies have shown that most scholars^17^, ^18^, ^19^ believe that the nature of the lesion is petechial bleeding, whereas other studies have suggested that it may be myelin destruction,^12^ Waller's degeneration,^20^ or “reversible calcium deposit and regurgitation.”^29^ With good control of blood glucose, chorea symptoms will be relieved soon, and the density of high‐density lesions in the striatum area on CT will gradually decrease and drop to the normal density range. However, the decrease of density on CT will be later than the disappearance of symptoms, and the change of MRI signal will be more delayed, later than the recovery of density on CT.^15^

The definition of DS was introduced about 10 years ago and is now probably the most complete and up to date. It describes a relatively uncommon condition of hyperglycemia associated with chorea/ballism and basal ganglia hyperdensity on CT and/or hyperintensity on T_1_‐weighted nuclear MRI. DS would also include patients with a hyperglycemic condition associated with even one of the following: (a) chorea/ballism or (b) striatal hyperdensity on CT or hyperintensity on T1‐weighted MRI.^30^ The term “diabetic striatopathy” is ambiguous and controversial. Furthermore, a possible classification of DS has been recently proposed, including symptomatic DS (striatal neuroimaging lesions in association with a clinically evident movement disorder and hyperglycemia), clinically isolated DS (clinically evident movement disorders without striatal changes in neuroimaging), and radiologically isolated DS. Radiologically isolated DS means that striatal changes in brain imaging associated with hyperglycemia (after exclusion of all probable etiologies having the potential to cause similar changes) without any evidence of movement disorders clinically, just like case 4 in this paper. Of interest is that 2% of patients may show radiological striatal lesions but no clinically manifested movement disorders (radiologically isolated DS).^31^

Current studies on the pathogenesis of hemichorea associated with nonketotic hyperglycemia suggest a metabolic disorder theory^32^: When considering high blood glucose, local cerebral blood flow decreased and glucose metabolism failure. Insufficient brain cell energy metabolism, tricarboxylic acid circulation was depressed, γ‐aminobutyric acid is the main energy source of brain cells. Patients with ketosis can use acetoacetic acid to synthesize γ‐aminobutyric acid again, but patients with nonketosis high blood glucose do not have such ability, eventually γ‐aminobutyric acid quickly exhausted. However, dopamine function is relatively enhanced, which destroys the normal function of basal ganglia striatum and eventually produces chorea. On the other hand, autoimmune diseases are also related to chorea disease. Studies have shown that^33^ glutamic acid decarboxylase (GAD) is a marker of GABAergic neurons in the striatum. Anti‐GAD65 antibodies can be detected in almost all type 1 diabetes patients and about 10% of type 2 diabetes patients. When blood glucose increases the permeability of the blood–brain barrier, GAD65 antibody may be involved in the basal ganglia and cross‐react with susceptible neurons, thus destroying the functional abnormality of the basal ganglia and also producing chorea symptoms. Hsu et al^34^ conducted PET examination on three patients and found that glucose metabolism in basal ganglia area on the diseased side was significantly reduced compared with that on the contralateral side, which could support this theory from another perspective. In addition, the dancing symptoms were quickly relieved by active blood glucose control without the use of dopamine blockers or sedatives, which seems to be better explained by the theory of metabolic disorders. Among the five patients treated in our hospital, four patients also showed obvious improvement in symptoms under blood glucose control, which was consistent with the preceding discussion. In addition, the motor circuit of the basal ganglia is the main part that controls the normal movement of the human body. When the balance between the direct pathway and the indirect pathway is broken, various motor disorders will occur, among which, the excessive inhibition of the indirect pathway causes chorea.^35^ So that anything that causes basal ganglia damage can cause chorea. For diabetic patients, it is more likely to cause or aggravate the insufficiency of blood supply or lacunar infarction in the basal ganglia area, thus causing damage to the indirect pathway of inhibiting the basal ganglia area and producing chorea symptoms.^36^ As a number of reports have proposed, the incidence of this disease in women is higher than that in men, so we cannot exclude hormone‐related effects. It has been reported in the literature that estrogen may inhibit the function of dopaminergic neurotransmitters in the substantia nigrostriatum system. Because estrogen levels in women are significantly reduced after menopause, this inhibitory effect will be somewhat weakened, and the sensitivity of dopamine receptors in the striatum will increase, ultimately enhancing the function of dopamine.^37^ It is for this reason that in the treatment of this disease, dopamine blockers can be used with good results. But this female predominance was also debatable. Dubey reported about the movement disorders that among the 59 patients, 52.5% were men.^38^ Results in another study showed that 36.36% (*n* = 4) of patients were female, and 63.63% (*n* = 7) were male.^39^ There is also a theory that female predominance is probably due to underdiagnosis in males rather than a real gender difference.^40^ In addition to the aforementioned possible mechanisms, other scholars have reported that hyperglycemia may cause metabolic disorders of peripheral blood cells to form spinous erythrocytes to a certain extent, so as to cross‐react with antigens and antibodies in the striatum, participating in the pathological process of chorea,^41^ which is also a possible pathogenesis. From the treatment aspect, hypoglycemic therapy and dopamine inhibitors are the main methods. There may be some other methods that can alleviate the symptoms but we still do not know why. Recently, Chen^42^ has reported one male patient, aged 59 years, who had a history of type 2 diabetes and poor blood glucose control, diagnosed with NH‐HC due to involuntary movements of the right limb and imaging findings from MRI. Except for glycemic control, acupuncture treatment at “governor vessel 13 acupoints” was beneficial for treatment of NH‐HC, and the involuntary movements completely disappeared on the ninth day of hospitalization. Besides, Globus pallidus internus deep brain stimulation is an effective and safe treatment option for hyperkinetic movement disorders secondary to brain tissue damage caused by hyperglycemia.^43^ This remedy had been reported in a 62‐year‐old male patient with a 28‐year history of type 2 diabetes with NH‐HC.

In patients with chorea, it is necessary to exclude other common diseases that cause involuntary movement of the hemi‐limb and combine blood glucose and ketone body levels to make the diagnosis, so it is very important for the differential diagnosis of this disease. For the treatment of this disease, the most important thing is etiological treatment, and active blood glucose control is crucial.If the patient's clinical symptoms can be properly combined with dopamine receptor antagonists and sedative drugs, this may have auxiliary effect on the remission of the disease. The clinical symptoms of patients can gradually disappear with the stable blood glucose, the rapid ones can be relieved within a few days, and the slow ones may need a few weeks. Only a few cases have the recurrence of the disease or symptoms, most of which are still related to the poor control of blood glucose, and the overall prognosis is good. For clinicians, how to quickly and accurately identify this disease in clinical work and treat it as soon as possible is the top priority.

## FUNDING INFORMATION

This project was supported by the National Natural Science Foundation of China (Grant No. 82000763) and the Tianjin Municipal Health Commission (Grant No. KJ20009).

## CONFLICT OF INTEREST STATEMENT

The authors have no conflicts of interest.
